# Acquired immunodeficiency syndrome/human immunodeficiency virus knowledge, attitudes, and practices, and use of healthcare services among rural migrants: a cross-sectional study in China

**DOI:** 10.1186/1471-2458-14-158

**Published:** 2014-02-13

**Authors:** Ying Wang, Christopher Cochran, Peng Xu, Jay J Shen, Gang Zeng, Yanjun Xu, Mei Sun, Chengyue Li, Xiaohong Li, Fengshui Chang, Jun Lu, Mo Hao, Fan Lu

**Affiliations:** 1 Research Institute of Health Development Strategies, Fudan University, 130 Dong An Road, Shanghai 200032, China; 2 Department of Health Care Administration and Policy, School of Community Health Sciences, University of Nevada, 4505 Maryland Parkway, Las Vegas, NV, USA; 3 National Center for AIDS Control and Prevention, China Center for Disease Control and Prevention, Beijing 102206, China

**Keywords:** China, AIDS/HIV, Knowledge, Attitude and Practice (KAP) study, Rural migrant workers, High-risk sexual behavior, Health services use

## Abstract

**Background:**

Today’s rapid growth of migrant populations has been a major contributor to the human immunodeficiency virus (HIV) epidemic. However, relatively few studies have focused on HIV/acquired immunodeficiency syndrome (AIDS)-related knowledge, attitudes, and practice among rural-to-urban migrants in China. This cross-sectional study was to assess HIV/AIDS-related knowledge and perceptions, including knowledge about reducing high-risk sex.

**Methods:**

Two-phase stratified cluster sampling was applied and 2,753 rural migrants participated in this study. An anonymous self-administered questionnaire was conducted in Guangdong and Sichuan provinces in 2007. Descriptive analysis was used to present the essential characteristics of the respondents. Chi-square test and multiple logistic regression models were performed to examine the associations between identified demographic factors and high-risk sex, sexually transmitted disease (STD) symptoms, and access to HIV screening services among the seven types of workers.

**Results:**

58.6% of participants were knowledgeable about HIV/AIDS transmission, but approximately 90% had a negative attitude towards the AIDS patients, and that 6.2% had engaged in high-risk sex in the past 12 months. Logistic regression analysis revealed sex, marital status, income, migration and work experience to be associated with high-risk sex. Among the 13.9% of workers who reported having STD symptoms, risk factors that were identified included female gender, high monthly income, being married, daily laborer or entertainment worker, frequent migration, and length of work experience. Only 3% of migrant workers received voluntary free HIV screening, which was positively associated with monthly income and workplace.

**Conclusions:**

HIV/AIDS knowledge, attitudes, and practices among rural migrants in China remain a thorny health issue, and use of healthcare services needs to be improved. Low levels of education and knowledge regarding HIV/AIDS among housekeepers and migrant day laborers result in this population likely being engaged in high-risk sex. Government programs should pay more attention to public education, health promotion and intervention for the control of the HIV/AIDS epidemic in China.

## Background

More than 1.8 million people died of acquired immune deficiency syndrome (AIDS) throughout the world in 2012, and prevention and control of AIDS/human immunodeficiency virus (HIV) remain challenging. It has been reported that there were 34 million HIV-infected people worldwide in 2011 [1]. The AIDS/HIV epidemic has worsened in China in recent years. The United Nations, World Health Organization, and Ministry of Health of China Joint Panel on HIV and AIDS estimated that HIV-positive cases in China reached 780,000 in 2011 [2]. Among 34,000 AIDS patients registered in the National AIDS Epidemic Reporting Network in 2010, 55.0% were infected through heterosexual activities. Among them 68.7% contracted the disease through casual or commercial sexual interactions [2]. Because the source of the disease in China is more often through sexual transmission than through blood transfusion, control of the HIV infection epidemic in China seems to be much more difficult.

The increase in the migrant population is one of the major contributing factors in HIV spreading in China [3,4]. In 2007, 79.2% of the HIV-positive were migrants [5]. According to the Sixth Census, there were more than 260 million migrant people in China, accounting for nearly 20% of the total population [6]. Most of China’s migrants were individuals who move from rural areas to urban areas for economic reasons such as better employment possibilities [7]. However, because they have no permanent urban household registration, migrants are marginalized and get no access to the social welfare benefits which are available to urban residents, such as subsided housing and long-term employment contracts [8]. Moreover, most of the migrants were young adults and they were more vulnerable to HIV exposure and infection due to the factors that increase their risks of pursuing sexual activities, and these factors include their little education, inadequate knowledge of safe sex, not being educated in health prevention practices, being in a new environment and separated from families [9-11]. Previous research suggests that employment-related factors may contribute to unsafe sex practices and that self employed individuals were more likely to engage in unprotected sex with casual sex partners [12]. Because of their frequently job changes and living place relocation, they have become the so-called “bridge population” that in HIV spreading [13].

Numerous studies of AIDS/HIV-related knowledge, attitude, and practice (KAP) and voluntary counseling and testing (VCT) among rural migrant populations have been conducted in recent years, but most them have focused on construction workers [14]; factory workers [15]; street vendors [16]; entertainment workers [17]; and catering or hotel servers [18]. Based on our literature review, no study has yet targeted two rural migrant population subgroups: day laborers (e.g., sewer dredge workers, housing renovation workers, and painters) and domestic workers. These two types of jobs have similar characteristics, such as temporary, low-pay, labor-intensive, and without insurance coverage, which often leads to frequent job changing. Furthermore, these two types of workers are loosely organized and offering health education programs to them is understandably difficult [19,20]. We included these two groups of workers in this study to make our study sample more representable among migrants.

Definitions of risky sex also differ within the AIDS/HIV-related literature. Some researchers regard “having sex with more than one person in one’s life” as risky sex [21], while others define risky sex as “having more than one stable sexual companion or had more than one casual sex partner, having prostitution-related sex or male homo sex, in the past 12 months” [22]. Neither view, however, considers the protective effect of condom use during sex. Studies have shown that continuous and correct use of condoms during sex can achieve safe sex up to 90%–95% of the time and also reduce the risk of AIDS/HIV infection by 10–20 times compared with the risk when not using a condom [23]. This study defines risky sex as all unprotected sexual behaviors [24]. Among married couples, sex without using condoms is considered risky if one of the partners also participates in unprotected extramarital sex; in particular, with casual or commercial sex partners. We believe that this definition is more understandable and accurate.

China is facing great challenges in achieving the United Nations’ goal of “providing universal access to comprehensive services (i.e., prevention, treatment, care and moral support) on AIDS/HIV by 2010 and containing the AIDS/HIV epidemic by 2015” [25]. This study, by targeting two provinces with large migrant populations, aimed to further understand AIDS/HIV-related KAP and use of related healthcare services among seven types of migrant populations. Specifically, we attempted to identify factors that are associated with high-risk sex and then to identify effective intervention strategies for AIDS/HIV prevention and screening. Given the large size of the study population, findings of the study have important implications for public health and policy.

## Methods

### Study population

A cross-sectional survey on AIDS/HIV-related KAP and use of health services among migrants was conducted in Guangdong and Sichuan province. Guangdong had the largest migrant population in China, 16.0 million, in 2007 [26]. In the same year, Sichuan had the largest migrant population in western China, 9.8 million [27]. We defined a migrant as one who was born and registered as a permanent resident in a rural area but has resided in an urban area for more than 3 months, or one who has resided in an urban area for less than three months but left his/her hometown in a rural area more than three months ago. They were also called “rural-to-urban migrants” we short-named them “migrants” in this study. Seven types of migrant workers covered the majority of migrants were surveyed. They were construction workers, factory workers, street vendors, entertainment workers, catering/hotel servers, day laborers, and domestic workers.

### Sampling

A strict pre-survey was conducted at two sampling metropolitan areas, Guangdong and Sichuan in 2007. It was found that the ratio of seven groups was near 0.4 (domestic servers): 1:1:1:1:1:1. We calculated out that 218 workers in six work sites and 50 domestic servers were needed in each of the two sampling metropolitan areas [28]. In other words, 1,358 were needed in each area for a total of 2,716 for the two provinces. The survey was conducted from April 2007 to December 2007.

### Data collection

An anonymous questionnaire was administered to obtain information regarding a subject’s socio-demographic characteristics (age, sex, monthly income, education level, marital status, whether living with spouse or not, and workplace), migration experience (number of cities ever lived in, number of years as a migrant worker), AIDS-related KAP (eight questions about HIV transmission channels, attitude toward AIDS patients, and risky and high-risk sex), sexually transmitted disease (STD) symptoms, and AIDS/HIV-related healthcare services use (healthcare seeking and screening). Written informed consent was obtained from all participants in Shanghai. This study was approved by the Fudan University Research Ethics Board. The approval number is IRB#2013-03-0444.

A strict protocol was followed during the implementation of the survey. Agreements were signed between local governments and the research team’s university. Permission was requested from employers to allow cooperation while interviewing employees. Official documents were issued by local governments to ensure employers’ active collaboration. The anonymity of the survey was achieved by de-identifying participants. Local dialects were used to make questions easier to understand. All the participation was based on informed consent and voluntary. A 100 yuan value of gift (i.e., cooking oil, bus card, or other gifts that valued 100 yuan) was given to each participant.

### Measures

The eight AIDS/HIV-related questions in our survey were: (1) Can an HIV-infected person be recognized from his/her appearance? (2) Can mosquito bites spread HIV? (3) Can having meals with HIV carriers get you infected? (4) Can receiving HIV-infected blood get you infected? (5) Can sharing syringes with HIV carriers get you infected? (6) Would the child born to a woman with HIV become infected? (7) Can correct use of condoms reduce the risk of spreading HIV? (8) Can having sex with only one person reduce the risk of spreading HIV? We defined a dichotomous variable, with a value of “1” indicating that a participant had a good understanding of HIV transmission channels, which meant he or she answered at least six of the eight questions correctly.

For the purpose of this study, casual sex was defined as having sex with one who is not his/her stable partner. Commercial sex was defined as sex involving money or goods exchange between two parties. Risky sex meant either casual sex or commercial sex, and high-risk sex meant casual or commercial sex without using a condom.

Finally, we defined several self-reported symptoms related to STDs given that lab results were not available. Studies have shown that although discrepancies may exist between self-reported STD infections and actual STD diseases, they are highly correlated [29]. After consulting with a panel of STD experts, we identified 11 STD-related symptoms, three for men and eight for women. The three symptoms for men were micturition pain or burning sensation, abnormal urethral discharge, and genital skin damage or proliferation. The eight symptoms for women were increased vaginal discharge and odor, pruritic vulvae, vaginal pain or burning sensation, non-menstrual lower abdominal pain, perineal skin damage, appearance of genital vegetation, urgent, painful, or more frequent urination, and dyspareunia.

### Data analysis

We used Epidata 3.02 for data entry and SPSS 17.0 for data analysis. Descriptive analyses were conducted to illustrate the KAP and use of AIDS/HIV-related healthcare services, and the chi-square test was conducted to examine significant differences between subgroups. Three multiple logistic regression models were then created to identify factors associated with high-risk sex, STD symptoms, and use of HIV screening services among the seven types of workers, respectively. In the first model, the dependent variable was “having high-risk sex in the last month” and the independent variables included age, sex, monthly income, education level, marital status, workplace, number of cities ever lived in, number of years as a migrant worker, AIDS/HIV knowledge, and attitude toward risky sex. In the second model, the dependent variable was “having had any of the STD symptoms,” and we added the independent variable “having high-risk sex” to all variables in the first model. In the third model, the dependent variable was VCT use, and we added the variable “having had any of the STD symptoms” to the variables in the second model.

## Results

Table 1 displays socio-demographic characteristics of rural migrants in our study by occupational clusters. Overall, the ratio of men to women was close to 1:1, their mean age was 31.3 years old. the average income was about 1,200 yuan (RMB), and 75% had an educational level of middle school or lower. The majority of participants (65.2%) were married, and nearly 40% of those were not living with their spouses. On average, they have migrated for 8.3 years and have been to 2.5 cities.

**Table 1:** Socio-demographic characteristics of study participants by working venues (%)

**Characteristics**	**Domestic servers**	**Factory workers**	**Street vendors**	**Catering/hotel server**	**Construction workers**	**Day laborers**	**Entertainment workers**	**Total**
Total respondents	106	448	432	441	448	435	443	2753
Age (year)^a^								
< = 24	8.5	35.7	34.3	65.5	8.0	7.6	59.1	34.0
25 ~ 34	20.8	34.6	28.9	22.7	25.4	35.4	32.7	29.6
35 ~ 44	54.7	23.2	26.2	8.8	40.4	44.6	5.4	25.9
45~	16.0	6.5	10.6	2.9	26.1	12.4	2.7	10.5
Sex^b^								
Male	13.2	52.7	39.4	40.8	92.6	74.3	18.3	51.5
Female	86.8	47.3	60.6	59.2	7.4	25.7	81.7	48.5
Monthly income (yuan)^a^								
<1000	92.5	48.4	61.4	77.1	42.5	81.3	44.9	60.4
1000 ~ 1500	6.6	33.9	16.2	14.5	29.2	14.3	22.1	21.2
> = 1500	0.9	17.6	22.5	8.4	28.3	4.4	33.0	18.4
Education level^a^								
Primary school or lower	34.9	12.5	17.2	5.2	35.9	33.3	9.7	19.6
Middle school	53.8	61.2	55.2	57.1	49.3	52.4	60.0	55.8
High school or above	11.3	26.3	27.6	37.6	14.7	14.3	30.3	24.6
Marital status								
Unmarried	8.5	35.3	32.6	64.2	14.3	16.1	52.4	34.8
Married	91.5	64.7	67.4	35.8	85.7	83.9	47.6	65.2
Live with spouse^b^	96	290	289	154	373	354	202	1758
Yes	59.4	67.9	86.2	61.7	42.4	54.8	58.9	60.8
No	40.6	32.1	13.8	38.3	57.6	45.2	41.1	39.2
Migrant cities^a^								
0	58.5	53.1	51.6	55.3	30.6	36.8	49.7	46.6
1	21.7	25.9	22.9	22.9	15.0	21.8	26.4	22.4
> = 2	19.8	21.0	25.5	21.8	54.5	41.4	23.9	30.9
Work years^a^								
<=6	43.4	49.8	45.8	68.0	30.8	36.1	65.5	49.1
>6	56.6	50.2	54.2	32.0	69.2	63.9	34.5	50.9
AIDS/HIV knowledge score^a^								
0-5	56.1	38.3	39.3	38.6	49.7	43.2	37.1	41.4
6-8	43.9	61.7	60.7	61.4	50.3	56.8	62.9	58.6
Work with AIDS patient^a^								
Unwilling	65.3	45.2	45.8	41.9	60.2	61.9	42.8	49.8
Willing but have no touch	23.5	30.9	27.7	30.0	21.5	24.3	29.6	27.4
Willing	11.2	19.4	21.2	26.2	16.5	11.9	23.9	19.8
Confused	0.0	4.4	5.3	1.9	1.8	1.9	3.6	3.1
Commercial sex^a^								
Agree	0.0	1.8	0.5	2.6	4.6	2.1	3.6	2.4
Disagree	84.8	75.5	76.6	75.3	62.8	72.5	58.1	70.9
Not care about	15.2	22.7	23.0	22.1	32.6	25.4	38.3	26.7
Casual sex^a^								
Agree	0.0	4.9	3.9	11.4	6.7	6.2	9.1	6.7
Disagree	84.6	65.2	62.0	57.0	55.7	66.6	54.7	61.3
Not care about	15.4	29.9	34.1	31.6	37.6	27.2	36.3	32.0

Note: ^a^means P < 0.01; ^b^means P < 0.05.

With regard to AIDS/HIV-related knowledge, 58.6% of the subjects were identified as having a good understanding of the disease. Entertainment workers had the best knowledge (62.9%) and domestic workers had the poorest (43.9%) In terms of respondents’ attitudes toward HIV-infected individuals, about half of the participants were unwilling to work with the people with HIV Among the seven types of jobs, relatively speaking, a low percentage of entertainment workers disagreed with commercial sex (58.1%) or casual sex (54.7%) whereas domestic workers had the highest percentages (84.8% for commercial sex and 84.6% for casual sex, respectively) (Table 1).

Table 2 shows descriptive results of risky sex. 165(6.0%) participants had commercial sex and 211(7.7% ) had casual sex in past 12 months The highest percentages among the seven types of workers for commercial sex were day laborers (13.6%) and entertainment workers (11.1%). As for casual sex, entertainment workers had the highest percentage (20.2%). A small number of participants (67 or 2.4%) engaged reported promiscuous sexual behavior with both prostitutes and other sexual partners. Moreover, 46.6% of participants who had sexual activities with prostitutes in the past 12 months did not use condoms each time, and among them, only 22.6% used a condom every time while having sex with their spouses or significant others. These two rates were 67.8% and 31.5% among those who had casual sex, respectively. A total of 170 respondents (6.2%) reported having high-risk sex, including 31.8% of entertainment workers, 27.6% of day laborers, and only 0.6% of domestic workers (Table 2).

**Table 2:** Sexual behaviors among study participants by working venues (%)

**Sexual activities characteristics**		**Domestic servers**	**Factory workers**	**Street vendors**	**Catering/hotel server**	**Construction workers**	**Day laborers**	**Entertainment workers**	**Total**
Commercial sex^a^	Respondents	106	448	431	441	447	435	440	2748
	Yes (N %)	1(0.9)	7(1.6)	8(1.9)	5(1.1)	36(8.1)	59(13.6)	49(11.1)	165(6.0)
	Condom use (%)								
	Never	0	28.6	0	20	19.4	32.8	10.4	21.1
	Occasional	100	0	50	20	22.2	24.1	29.2	25.5
	Every time	0	71.4	50	60	58.3	43.1	60.4	53.4
Casual sex^a^	Respondents	106	447	431	440	447	435	440	2746
	Yes (N %)	2(1.9)	10(2.2)	11(2.6)	29(6.6)	25(5.6)	45(10.3)	89(20.2)	211(7.7)
	Condom use (%)								
	Never	50.0	22.2	20.0	17.2	62.5	47.7	18.9	30.3
	Occasional	0	44.4	30.0	48.3	33.3	34.1	37.8	37.5
	Every time	50.0	33.3	50.0	34.5	4.2	18.2	43.3	32.2
High-risk sex^a^	Yes (N %)	1(0.6)	9(5.3)	9(5.3)	19(11.2)	31(18.2)	47(27.6)	54(31.8)	170(100.0)

Note: ^a^means P < 0.01.

Descriptive results of STD symptoms and related health services use are shown in Table 3. Entertainment workers had the highest percentage (27.1%). Only 43.9% of those who reported STD symptoms sought formal health care at hospitals, special clinics, or health centers, and 18.5% did not seek any care at all. Day laborers reported the highest percentage of using free condoms (12.4%). Nearly one quarter of respondents reported receiving free STDs/AIDS/HIV educational materials, 32.7% of whom were entertainment workers and only 13.2% of whom were domestic workers. In addition, only 2.9% of respondents received free HIV tests and 1.2% received counseling before the test. Finally, among those who had engaged in high-risk sex, 24.1% reported STD-related symptoms, compared with only 13.3% among those who did not engage in high-risk sex.

**Table 3:** STD-related symptoms and AIDS-related services use of study participants

**Characteristics**	**Domestic servers**	**Factory workers**	**Street vendors**	**Catering/hotel server**	**Construction workers**	**Day laborers**	**Entertainment workers**	**Total**
STD-related symptoms^a^ (N)	106	447	423	441	448	434	442	2741
Yes (%)	15.1	14.3	9.5	13.2	5.4	13.8	27.1	13.9
Medical care seeking behavior (N)	16	64	40	58	24	60	120	382
Hospital	25.0	42.2	35.0	32.8	29.2	26.7	33.3	33.2
Special clinics	6.3	4.7	5.0	10.3	4.2	1.7	5.8	5.5
Health centers	31.3	14.1	22.5	12.1	0.0	5.0	19.2	14.7
Private clinics	12.5	12.5	17.5	10.3	16.7	28.3	22.5	18.6
Self-treatment	37.5	25.0	25.0	27.6	33.3	35.0	27.5	28.8
Do nothing	12.5	15.6	15.0	27.6	16.7	25.0	15.0	18.6
Intervene receiving situation (N)	106	448	432	441	448	435	443	2753
Condom distribution^a^	4.7	11.6	6.7	6.3	8.3	12.4	12.0	9.4
Educational materials^a^	13.2	21.0	22.7	25.4	19.0	25.7	32.7	24.0
Free HIV tests	0.9	2.9	1.4	1.4	2.7	0.7	8.8	2.9
Consultation	0.9	0.9	0.2	0.2	1.3	0.0	4.3	1.2

Note: ^a^means P < 0.01.

Table 4 lists the results of multiple logistic regression that examined factors associated with high-risk sex. Respondents who were male or/and unmarried, had a higher monthly income, were frequent migrants, and had worked more years were more likely to engage in high-risk sex.

**Table 4:** Logistic regression analysis of factors correlated with high-risk sex

**Variables**	**Control group**	**Comparable group**	**P value**	**Exp (B)**	**95% CI for EXP (B)**	
					**Lower**	**Upper**
Sex^a^	Female	Male	0	2.956	1.86	4.697
Monthly income^b^	<1000		0.015			
		1000-1500	0.609	1.126	0.714	1.777
		> = 1500	0.005	1.919	1.221	3.017
Marital^a^	Married	Unmarried	0	3.888	2.478	6.1
Work venue^a^	Domestic servers		0			
		Factory workers	0.972	0.962	0.114	8.143
		Street vendors	0.86	1.21	0.145	10.097
		Catering/hotel servers	0.513	2	0.251	15.967
		Construction workers	0.38	2.522	0.32	19.891
		Day laborers	0.117	5.101	0.664	39.169
		Entertainment workers	0.057	7.336	0.943	57.054
Migrant cities^b^	0		0.004			
		1	0.017	1.808	1.114	2.934
		> = 2	0.001	2.288	1.396	3.752
Work years^b^	< = 6		0.005			
		>6	0.002	1.869	1.249	2.798
Constant^a^			0	0.002		

Note: ^a^means P < 0.01; ^b^means P < 0.05.

Table 5 shows the results of multiple logistic regression analysis on factors associated with STD-related symptoms and receipt of AIDS/HIV screening. The odds of females having STD symptoms were nine times as much as those of their male counterparts (odds ratio (OR) [95% confidence interval (CI)], 9.00 [6.24, 12.95]). Respondents who engaged in high-risk sex were more likely to have STD symptoms than those who did not (OR [CI], 2.87 [1.83, 4.51]). Higher monthly income, being married, day laborers, and entertainment workers were associated with STD symptoms. Further, monthly income and workplace were associated with free HIV screening. Compared with domestic servers, entertainment workers were more likely to have free HIV screening (OR 10.26 [CI 2.81, 37.46]).

**Table 5:** Logistic regression analysis of factors correlated with STD-related symptoms and free HIV test

**Variables**	**Control group**	**Comparable group**	**P value**	**Exp (B)**	**95% CI for EXP (B)**	
					**Lower**	**Upper**
STD-related symptoms						
Sex^a^	Female	Male	0	8.988	6.237	12.952
Monthly income^b^	<1000		0.002			
		1000-1500	0.129	0.766	0.544	1.08
		> = 1500	0.015	1.503	1.083	2.086
Marital status^b^	Unmarried	Married	0.006	1.643	1.15	2.349
Work venue^a^	Domestic servers		0			
		Factory workers	0.117	1.661	0.881	3.133
		Street vendors	0.34	0.725	0.375	1.403
		Catering/hotel servers	0.312	1.401	0.729	2.692
		Construction workers	0.473	1.323	0.616	2.838
		Day laborers	0.003	2.665	1.391	5.104
		Service staffs	0.04	1.946	1.032	3.669
High-risk sex^a^	No	Yes	0	2.869	1.826	4.51
Constant^a^			0	0.011		
Free HIV test						
Monthly income^b^	<1000		0.013			
		1000-1500	0.003	2.363	1.328	4.206
		> = 1500	0.104	1.675	0.899	3.121
Work venue^a^	Domestic servers		0			
		Factory workers	0.074	6.517	0.833	50.984
		Street vendors	0.002	3.106	1.495	6.45
		Hospitality workers	0	6.193	2.436	15.742
		Construction workers	0	5.337	2.13	13.376
		Daily laborers	0.007	3.527	1.417	8.777
		Entertainment workers	0	10.256	2.808	37.463
Constant^b^			0.002	7.333		

Note: ^a^means P < 0.01; ^b^means P < 0.05.

## Discussion

This study improves our understanding of AIDS/HIV-related KAP of migrant populations in China, especially day labors and domestic workers who have been studied in prior research. Our findings indicate that as predominant males with frequent migrating experience and long working years, day laborers tend to engage in high-risk sex but also use condoms as compared to the rest of the six types of workers except for entertainment workers. On the other hand, most domestic workers are married women who have very limited general education and little knowledge about AIDS/HIV. Their acceptance level for commercial and casual sex, as well as of high-risk sex and condom use, was low. Our findings also indicate that day laborers and domestic workers have similar percentages of self-reported STD symptoms, which were close to the average of the seven types of migrant populations being focused in our study.

Further, our findings indicate that people with good knowledge of AIDS/HIV may not necessarily avoid having risky sex. Due to strong governmental campaigns on AIDS/HIV prevention, general public, including migrants, become more knowledgeable about the disease but may not alter their risky sexual behavior (so called “the knowing-doing gap”). It should be noted that those with good knowledge do tend to use condoms, which reduces the risk associated with STDs. Given that completely eliminating risky sex does not seem to be possible, at least in the short run, focusing more attention on encouraging condom use is a feasible and effective approach to discouraging high-risk sex and preventing the spread of AIDS/HIV [30]. Our findings confirm that a greater number of day laborers were more likely to use free condoms and, therefore, suggest that the provision of free condoms to other migrant workers can have the same benefits. However, our findings of condom use among commercial and casual sexual encounters are slightly lower than that found in other studies [31] and far below the national goal of 100% [32,33]. The percentage of migrants receiving free condoms in our study was also lower than that in other studies [34-36]. Since it takes times to transfer knowledge and attitude to behavioral changes, more effective intervention approaches are merited for future research.

Our findings indicate that the migrant populations are at sexually active ages with [37] relatively low education levels and income. These are consistent with previous studies that reported that mobility is a key determinant of HIV/sexually transmitted infection (STI) [38]. Our findings are also consistent with those of other studies that migrants in China have high rates of unprotected extramarital sex and unprotected marital sex [39,40]. In addition, high-risk sex practices are correlated with frequent migrations and years of working as migrants [19,41], and findings show that migrant workers in this study have quite long migrant experience. The combination of having unprotected sex, frequent migrations among cities, and back-and-forth travels between cities of their employment and their hometowns, migrants might become a bridge population for HIV and sexually transmitted infections (STIs) [15,42].

The percentage of migrant workers having a good understanding of AIDS/HIV transmission in our study is below the national goal that is “70% of migrants who are between 15 and 49 years old have good knowledge of AIDS/HIV transmission” [43]. The majority of migrant workers in our study also had a negative attitude toward HIV-infected individuals, which implied that the discrimination for AIDS patient still commonly existed. Women who engaged in high-risk sex, as well as day laborers and entertainment workers tended to report STD symptoms. One of explanations may be that with limited education, many migrant women lack knowledge of healthy sexual lifestyles, including proper use of contraceptives, which results in abortions and preventable STD infections [44]. Risks increase for female entertainment workers who tend to be poor and are more likely to accept higher pays for unprotected sex [45]. Although the percentage of migrant workers who reported STD symptoms but did not seek healthcare in our study is lower than those reported in other studies [46], the avoidance of health care among migrants is common. This reduces the opportunity to identify STDs among this population [47]. Since migrant workers are not registered as permanent residents in urban areas, they are not eligible for urban social health insurance programs. With relatively low income, they may not seek care for STD infections because of financial concerns.

Our percentage of migrant workers who received voluntary free HIV screening was only slightly higher than those reported in earlier studies [48]. Among workers who received such screening, less than a half also received pre-screening counseling. Among different types of migrant workers, entertainment workers seem to avail themselves of screening. This is probably because this subgroup has been the targeted population of several governmental public health intervention programs in recent years. In the realization that the first step to control the spread of HIV is to know whether one is infected [49], the Chinese government built 4,293 health services clinics/stations in all 31 provinces and equivalent regions by 2007 to provide free VCT screenings and counseling. Finally, our study further confirmed that lack of knowledge and negative attitudes would result in poor use of VCT, as mentioned in other studies [50].

## Conclusions

This study has limitations. Because it was a cross-sectional study, causal inferences cannot be drawn. Secondly, the survey contained many sensitive questions, such as those regarding risky sex. Given the traditional, and conservative culture of sexuality in China, response bias may factor into under-reporting of some of the information in this study. Recall bias may be a factor in the survey as well. Given the time lapse, some individuals may have provided information that was not completely accurate. Finally, information on STDs was self-reported and the accuracy of this self-reported diagnosis may be an issue without confirmation of laboratory tests.

In conclusion, migrants are at particularly high risk of HIV infection in China, as they frequently face marginalization, exclusion and various barriers to accessing health promotion and care. As a result, effective prevention of AIDS/HIV transmission among the migrant population remains challenging. Experts in China believe that the key strategies for control of the AIDS/HIV epidemic should focus on public education and behavior-changing interventions [51]. However, based on the results of this study, we would recommend new intervention programs for migrant populations: (1) Provide proper educational materials that are appropriate to migrant workers’ education levels and offer education programs in various forms (e.g., peer education versus sexual partner engagement) at appropriate times and places (e.g., work sites); (2) Expand the distribution channels of free condoms and further publicize the importance of using condoms to prevent STDs to improve the acceptance of condom use and ultimately eliminate high-risk sex among migrant populations; (3) Strengthen the existing free VCT screening programs by making VCT information more accessible to migrant populations while providing more screening stations/clinics, from which the early detection and treatment as well as the secondary prevention will be improved [14,50]; and (4) Make greater efforts to provide treatment services including anti-retroviral therapy as recommend by the International Organization for Migration (IOM) which applied at early stage of AIDS to control HIV spreading [52].

## Abbreviations

AIDS: Acquired immune deficiency syndrome; HIV: Human immunodeficiency virus; KAP: Knowledge, attitude and practice; STD: Sexually transmitted disease; VCT: Voluntary counseling and testing.

## Competing interests

The authors declare that they have no competing interests.

## Authors’ contributions

MH and FL are Co-corresponding authors. MH is the first corresponding author and LF is the second one. MH mainly contributed to designing this research, revising the article and giving final approval of the version to be published, otherwise FL mainly contributed to designing this research and giving final approval of the version. YW the first author, was responsible for analyzing and interpreting the data, drafting the article and also giving final approval of the version to be published. PX and GZ who worked in China Center for Disease Control and Prevention are responsible for the research design and acquisition of data and also participated in revising the article and giving final approval of the version to be published. CRC, JJS especially critically revised and polished the article, and also contributed to designing the research and giving final approval of the version to be published. YX, MS, CL, XL, FC, JL participated in conceiving and designing the research, revising article and giving final approval of the version to be published as well. All authors read and approved the final manuscript.

## Pre-publication history

The pre-publication history for this paper can be accessed here:

http://www.biomedcentral.com/1471-2458/14/158/prepub

## References

[B1] AVERT Global Epidemic: Global HIV and AIDS estimates 2011 http://www.avert.org/worldwide-hiv-aids-statistics.htm

[B2] The Central People Government of the People Republic of China An estimate of 780,000 people living with HIV in China http://www.gov.cn/gzdt/2011-11/23/content_2001819.htm

[B3] Li XM Stanton B Fang XY Lin DH Mao R Wang J Cottrell L Harris C HIV/Std Risk Behaviors and Perceptions among Rural-to-Urban Migrants in China AIDS Educ Prev 2004 16 6 538 556 http://www.gov.cn/jrzg/2011-11/29/content_2006240.htm 10.1521/aeap.16.6.538.53787 15585430 PMC1791014

[B4] Wang Q On behavior intervention model of HIV prevention among floating population J Yunnan Police Officer Acad 2009 5 68 70

[B5] Li D Yan YP Liu JZ Lu SH Zhang L Su HX Knowledge on AIDS among the Transient Population in Shangluo Chin Gen Pract 2007 10 13 1092 1097

[B6] Ma JT Mainly datas of sixth national population census 2011 http://www.gov.cn/gzdt/2011-04/28/content_1854048.htm

[B7] Wang D Ye H Survey of study on the population migration in China after 1990 Population J 2004 1 40 46

[B8] Zhang KJ Zhou L Liang GJ Zhang JY Survey on knowledge, attitude and behavior about STD/AIDS among floating population China Public Health 2005 1 7 857 858

[B9] Yang LS Zhou JF HIV/AIDS prevention and health education problem discussed among young migrant population, taking jiangsu as an example J Nanjing Coll Popul Programme Manage 2002 18 4 9 11

[B10] Yang TZ Study on the dissemination of human immunodeficiency virus risk behaviors in a floating workers coming from the countryside in China Chin J Epidemiol 2006 27 3 264 269 16792905

[B11] Cheng X Zhang HZ Wang SJ Study on HIV/AIDS related behavior between migrant population and non-migrant population in rural area Chin Rural Health Serv Admin 2005 25 10 57 61

[B12] S-gM W Gao MY Employment and contextual impact of safe and unsafe sexual practices for STI and HIV: the situation in China Int J STD AIDS 2000 11 8 536 544 10.1258/0956462001916281 10990339

[B13] Tang HL LV F Role of bridge population in the transmission of human immunodeficiency virus Chin J Epidemiol 2007 28 2 192 194 17649696

[B14] Hu JF Chen YQ Ye JJ Survey on STD/AIDS knowledge, attitude among construction workers Zhejiang J Prev Med 2008 20 1 71 72

[B15] Zhang QH Lv F Population migration and AIDS/HIV transmission Prev Med Tribune 2006 12 3 216 218

[B16] Hu Z Wang DB Liu HJ STD/AIDS knowledge, attitude, practice, and characteristic of market vendors in Hefei, China AIDS Patient Care STDs 2005 19 2 121 126 10.1089/apc.2005.19.121 15716643

[B17] Chen XX Wei PM Huang MH Study on effects of multiple methods of health education on AIDS and forgetting of knowledge among floating population Mod Prev Med 2007 34 2 397 400

[B18] Zhang JL He N Socio-demographic characteristics and condom use among migrants in an area of central China Fudan University J Med Sci 2007 34 1 66 69

[B19] Liu CJ The Features and FP Management of Floating Population in China J Nanjing Coll Popul Program Manage 1998 14 3 6 9

[B20] Tian SG Zhang BS Informal employment of the migrant population-comparison between Shanghai and New York Shanghai Urban Development 2008 3 46 49

[B21] He N Detels R Chen Z Sexual behavior among employed male rural migrants in Shanghai, China AIDS Educ Prev 2006 18 2 176 186 10.1521/aeap.2006.18.2.176 16649962

[B22] Pan XH Yang JZ Xu Y Study on AIDS related risk sexual behaviors and influential factors among college students Chin J School Health 2008 29 8 701 703

[B23] Zhao SM Dong FF Li XM Study on sexual attitude and behavior among migrant workers J Nursing Sci 2008 23 7 1 4

[B24] Li XM Zhang L HIV/AIDS-related sexual risk behaviors among rural residents in China: potential role of rural-to-urban migration AIDS Educ Prev 2007 19 5 396 407 10.1521/aeap.2007.19.5.396 17967110 PMC2064039

[B25] United Nations General Assembly Special Session on HIV/AIDS Guidelines on Construction of Core Indicators 2010 New York, USA

[B26] Statistics Bureau of Guangdong Province Guangdong statistical yearbook 2008 [DB/OL] http://www.gdstats.gov.cn/tjnj/2008/ml_c.htm

[B27] Statistics Bureau of Sichuan Province Sichuan statistical yearbook 2008[DB/OL] http://jg.scjg.com.cn/company/mobon6/index.asp?ep_id=187

[B28] Li LM Epidemiology 2008 Beijing, China: People’s Medical Publishing House 44

[B29] Fang J Zhang KN Reproductive tract infection-Research on global current and dynamic situation 2002 Beijing, China: People’s Medical Publishing House 25 31

[B30] Du JW Nie SF Advance in research of prevalent factors of AIDS and intervention measures China Trop Med 2008 8 7 1249 1251

[B31] Lin DX Li ZJ Liu W Wei X Lv F Zeng G Analysis of HIV/AIDS prevention and control among mobile population in Hezhou, Guangxi Disease Surveillance 2008 23 6 364 364

[B32] He WJ Cui LX Research and analysis on knowledge, attitude and practice about STD/AIDS among migrant population in Nanyang Henan J Prev Med 2011 22 2 105 108

[B33] World Health Organization Regional Office for the Western Pacific Responding to questions about the 100% condom use program 2004 Geneve, Switzerland

[B34] Chen W Li Q Liu L Dong LM Lu B Song GR AIDS-related KAP and the related factors among migrant people in some middle-scaled tourism city Modern Prev Med 2010 37 3 502 504

[B35] Li Z Shi SH Ma JD Study on the demand of AIDS prevention and health promotion in the floating population Maternal Child Health Care China 2005 24 3213 3215

[B36] Wang M Hsieh J Ling YQ HIV/AIDS prevention and control of migrant population in China Strait J Prev Med 2004 10 2 66 67

[B37] The Central People Government of the People Republic of China General office of the national population and family planning commission on further notice for HIV/AIDS prevention work http://www.gov.cn/gzdt/2011-11/23/content_2001819.htm

[B38] Goldenberg SM Strathdee SA Perez-Rosales MD Sued O Mobility and HIV in Central America and Mexico: A critical review J Immigrant Minority Health 2012 14 48 64 10.1007/s10903-011-9505-2 21789558

[B39] Li B Wang Y Zang F Gao B YONG LZ WEI LQ Study on extramarital sexual intercourse and condom use related factors among floating population Chin J Public Health 2008 24 7 791 792

[B40] Xia DY Lu HY Liu GW Li GY Ma XY He X Wang N Prevalence of HIV infection, syphilis, HCV and risk behaviors among migrant population in Beijing Chin J AIDS STD 2011 17 4 430 432

[B41] He N Detels R Characteristics and sexually transmitted diseases of male rural migrants in a metropolitan area of Eastern China Sex Transm Dis 2005 32 5 286 292 10.1097/01.olq.0000152219.58592.9b 15849529

[B42] Kramer MA van Veen MG Op de Coul ELM Geskus RB Coutinho RA van de Laar MJW Prins M Migrants travelling to their country of origin: a bridge population for HIV transmission? STI Online First 2008 doi:10.1136/sti.2008.032094 10.1136/sti.2008.032094 18653565

[B43] The Chinese AIDS working committee office Supervision and evaluation framework for AIDS prevention and control in China (on trial) 2007 Beijing, China

[B44] Wu JQ Current status of sexual and reproductive health among migrants in China J Int Reprod Health/Family Planning 2010 29 6 414 421

[B45] Goldenberg SM Unpacking Mobility, Sex Trafficking, and HIV Vulnerability in Two Mexico- U. S. Border Cities 2011 Southern California San Diego: University of California, San Diego, San Diego State University

[B46] Wu Q He N Gu P Xu L Status quo of health service utilization of STD/HIV among migrant women in a district of Shanghai Chin J Health Educ 2007 23 7 518 520

[B47] Fu Y HIV/AIDS prevention and control for male migrant rural workers Mod Prev Med 2007 34 16 3176 3177

[B48] Li QH Zhu WL Tian XH Zhang TJ Ma FC He N Study on receptivity of floating population to HIV voluntary counseling and testing in a community of Shanghai City Chin J Health Educ 2009 25 8 586 589

[B49] World Health Organization Increasing access to HIV testing and counseling. Report of a WHO consultation, 19–21 November 2002 2003 Geneva, Switzerland http://www.who.int/hiv/pub/vct/pub36/en/index.html

[B50] Ma FC Yao JM Wang Y Yang Y Huang H Zhang JH A qualitative study on voluntary counseling and testing (VCT) for HIV/AIDS among migrants in Minhang district of Shanghai Chin J AIDS STD 2008 1 36 38

[B51] Zeng Y Public education and intervention is main strategy to control the HIV/AIDS epidemic J Fujian Univ Traditional Chin Med 2010 20 2 1 2

[B52] International Organization for Migration HIV and Population Mobility 2011 http://www.iom.int/cms/en/sites/iom/home/news-and-views/press-briefing-notes/pbn-2012/pbn-listing/world-aids-day-migrants-dispropo.html

